# Supplementary material for: Loss of Control over Eating, Inhibitory Control, and Reward Sensitivity in Children and Adolescents: A Systematic Review
Source: Nutrients. 2023 Jun 8;15(12):2673. doi: 10.3390/nu15122673 (PMC10303700; doi:10.3390/nu15122673)
Supplement: Supplementary file 1 [file nutrients-15-02673-s001.zip › nutrients-2437258-supplementary.pdf]

nutrients

MDPI

Supplementary Material

Table S1. Search strategy.

**Search**		**Search Queries**
#1	Title, Abstract and Keywords	adolescence OR adolescents OR child OR children OR youth
#2	Title, Abstract and Keywords	“executive function*” OR inhibition OR “inhibitory control” OR reward OR “reward sensitivity” OR impulsivity
#3	Title, Abstract and Keywords	“loss of control over eating” OR “loss of control eating” OR “uncontrolled eating” OR “dysregulated eating” OR “binge eating”
#4		#1 AND #2 AND #3
#5		#4, from 2000 to 2021 (February)
